# Hybrid Nanoparticles from Random Polyelectrolytes and Carbon Dots

**DOI:** 10.3390/ma17102462

**Published:** 2024-05-20

**Authors:** Sophia Theodoropoulou, Antiopi Vardaxi, Antonia Kagkoura, Nikos Tagmatarchis, Stergios Pispas

**Affiliations:** 1 Theoretical and Physical Chemistry Institute, National Hellenic Research Foundation, 48 Vasileos Konstantinou Avenue, 11635 Athens, Greece; sophiatheodor97@gmail.com (S.T.); avardaxi@eie.gr (A.V.); akagkoura@eie.gr (A.K.); 2 Department of Chemistry, National and Kapodistrian University of Athens, Panepistimioupolis Zografou, 15771 Athens, Greece

**Keywords:** polyelectrolytes, carbon quantum dots, hybrid nanoparticles, complexes, RAFT polymerization

## Abstract

The present study concerns the preparation of hybrid nanostructures composed of carbon dots (CDs) synthesized in our lab and a double-hydrophilic poly(2-dimethylaminoethyl methacrylate-*co*-oligo(ethylene glycol) methyl ether methacrylate) (P(DMAEMA-*co*-OEGMA)) random *co*polymer through electrostatic interactions between the negatively charged CDs and the positively charged DMAEMA segments of the *co*polymer. The synthesis of P(DMAEMA-*co*-OEGMA) *co*polymer was conducted through RAFT polymerization. Furthermore, the *co*polymer was converted into a strong cationic random polyelectrolyte through quaternization of the amine groups of DMAEMA segments with methyl iodide (CH_3_I), and it was subsequently utilized for the complexation with the carbon dots. The molecular, physicochemical, and photophysical characterization of the aqueous solution of the *co*polymers and their hybrid nanoparticles was conducted using dynamic and electrophoretic light scattering (DLS, ELS) and spectroscopic techniques, such as UV-Vis, fluorescence (FS), and FT-IR spectroscopy. In addition, studies of their aqueous solution using DLS and ELS showed their responsiveness to external stimuli (pH, temperature, ionic strength). Finally, the interaction of selected hybrid nanoparticles with iron (III) ions was confirmed through FS spectroscopy, demonstrating their potential application for heavy metal ions sensing.

## 1. Introduction

Over the last few years, carbon dots (CDs) have attracted much scientific interest. These materials are thought to be favorable for various biological and optoelectronic applications due to their beneficial attributes, like minimal toxicity, unique photoluminescence properties, and great chemical stability [1,2]. Numerous industries, such as energy conversion, food quality evaluation, and material science, have used CDs for a wide range of biological uses, such us bioimaging, biosensing, ion detection, and tracing of crime scenes [3]. As a new carbon allotrope, CDs are a promising material for a variety of applications due to their many unique qualities, which include reduced cytotoxicity, strong biocompatibility, sustained chemical inertness, efficient light harvesting, and exceptional photoinduced electron transfer, which permit their use in optoelectronic devices, biosensors, and bioimaging Ref. [4]. CDs are highly recommended as anode materials in electrochemical systems due to their exceptional flexibility and conductivity Ref. [5]. Furthermore, changes in the surface band bending brought on by nearby external agents (e.g., molecules) can strongly impact the intensity of their photoluminescence yield Ref. [6]. Recently, CDs and polymer composite materials have attracted a lot of interest for biochemical, biological, and medicinal applications due to their easy manufacturing, economical processing, and biocompatibility [7]. In this respect, a growing area of study is creating improved CD/polymer composite materials with beneficial optoelectrical and biocompatibility characteristics [3].

In this direction, the usage of random *co*polymers combined with CDs for novel nanocomposite materials appears as an intriguing prospect, yet little research has been conducted on the subject [8]. Random (or statistical) copolymers consist of monomers that are randomly distributed along the macromolecular chain. In this case, the properties of the component monomeric groups are combined to the ultimate polymer macromolecule [9]. Such *co*polymers can be easily synthesized via a single-step *co*polymerization of two or more monomers, such as the reversible addition–fragmentation chain transfer (RAFT) radical polymerization. Due to the variety of monomers that can be utilized in a controlled manner, the facile implementation, the low cost, and the capacity to create polymers with high molecular weights and narrow molecular weight distributions, RAFT polymerization is considered to be superior to other competitive methods of controlled radical polymerization, such as ATRP (Atom transfer radical polymerization) and NMP (Nitroxide-mediated polymerization) [10,11]. Nonetheless, extensive self-assembly experiments have been hampered by the challenge of creating well-structured morphologies with narrow dispersity utilizing random *co*polymers with wide molecular distribution [12]. Specifically, amphiphilic random *co*polymers, consisting of hydrophobic and hydrophilic monomers, present a particularly interesting behavior when dissolved in aqueous or organic solvents. The solvent’s nature defines the self-assembly behavior of the random *co*polymers, resulting in intramolecular self-folding into unimolecular nanosized micelle-like aggregates with hydrophobic domains [12,13,14]. On the other hand, double-hydrophilic random *co*polymers, wherein both components are soluble in water, have the property of behaving as amphiphiles under certain conditions that induce hydrophobicity or reduced solubility to one of their monomeric units [15].

In addition, stimuli-responsive *co*polymers are also gaining a lot of scientific interest due their large gamut of applications in industrial as well as biomedical fields [16]. This response can be identified by studying the effect of external stimuli on the self-assembly behavior of the polymer aqueous solution. More precisely, this characteristic relates to polymers that contain monomeric components with a tendency to respond to changes in pH, temperature, ionic strength, and many other chemical or physical stimuli. Polymers of this type are considered “smart” and ideal for encapsulation or complexation with a variety of other materials, owing to their ability to be influenced by external stimuli under certain circumstances [17]. As for the temperature factor, some polymers exhibit lower critical solution temperature (LCST), which is the lowest temperature that causes phase separation between the solution and the polymer [18,19,20]. In addition, polymers bearing ionized monomers can undergo similar structural changes in response to pH variations. These polymers usually carry acidic or basic functional groups and are known as polyelectrolytes. Because of the pH changes, the degree of ionization of the polymer shifts around a certain pH value, called pK_a_. Hence, the hydrodynamic volume of the polymer chains changes as a response to the abrupt shift in the macromolecule’s overall load [21,22]. Polymers carrying ionized monomers are also affected by ionic strength variations in the solution. The presence of salt ions leads to variations in the hydration profile around the polymer chains by reducing the phase separation temperature [23].

This work aims to present novel, hybrid nanostructures obtained through the complexation of citric-acid-based CDs with double-hydrophilic P(DMAEMA-*co*-OEGMA) random *co*polymer and its modified derivative Q_P(DMAEMA-*co*-OEGMA), acquired through quaternization. The precursor *co*polymer was synthesized via a one-step RAFT polymerization process and, subsequently, the tertiary amine groups of DMAEMA units were modified using methyl iodide, thus creating permanent cationic charges on the obtained *co*polymer. P(DMAEMA-*co*-OEGMA) was the random copolymer of choice due the pH- and thermo-responsiveness of connected DMAEMA segments, as in the case of PDMAEMA homopolymer [12,24]. As for the LCST of the PDMAEMA homopolymer, the change from a hydrophilic to a hydrophobic state occurs around 40–50 °C and is strongly affected by the molecular weight, pH, and salt content in each case. On the other hand, POEGMA is a hydrophilic-profiled non-ionic polymer made up of grafted hydrophilic oligo(ethylene glycol) side chains and a hydrophobic carbon–carbon backbone, providing stability to the system [25,26]. Hence, P(DMAEMA-*co*-OEGMA), Q_P(DMAEMA-*co*-OEGMA) as well as the obtained complexes with CDs were molecularly and physiochemically characterized, and their aggregation profile upon heating and at varying pH values and ionic strengths was thoroughly investigated. Both polymers’ physiochemical behavior and responsiveness to external stimuli were investigated at the initial state of this study as groundwork for the subsequent characterization of the hybrid complexes. To make a connection to potential applications, the hybrid complexes were utilized as a sensitive sensor for ferric ions in order to examine their potential in biosensor applications.

## 2. Materials and Methods

### 2.1. Materials

Monomers 2-(dimethylamino)ethyl methacrylate (DMAEMA, 98%) and (oligo ethylene glycol)methacrylate (OEGMA) (average Mn = 950 g·mol^−1^), purchased from Sigma Aldrich (Athens, Greece), were purified through a column filled with inhibitor removers 311340 and 311332 (Sigma Aldrich). 2,2′ -Azobis (isobutyronitrile) (AIBN), which was used as the radical initiator, was purified through recrystallization from methanol. 1,4-dioxane (99.8% pure), the solvent utilized for the reaction, was obtained from Sigma Aldrich and dried using molecular sieves. 4-Cyano-4-(dodecylsulfanylthiocarbonyl)pentanoic acid (CDP), as the CTA, methyl iodide (CH_3_I), citric acid, ethylenediamine, tetrahydrofuran (THF, ≥99.9% pure), and deuterated chloroform (CDCl_3_) were used as obtained from Sigma Aldrich (Greece). Dialysis tubing membranes (MEMBRA-CEL^®^, Lombard, IL, USA) from regenerated cellulose of MWCO 3500 and a diameter of 22 mm were purchased from SERVA, Heidelberg, Germany.

### 2.2. Synthesis of P(DMAEMA-co-OEGMA) Random Copolymer

The synthesis of poly(2-(dimethylamino)ethyl methacrylate-*co*-(oligo ethylene glycol)methacrylate) random copolymer was achieved via one-step reversible addition–fragmentation chain transfer polymerization (RAFT). Monomers were initially purified using a column filled with monomethyl ether hydroquinone (MEHQ) and butylated hydroxytoluene (BHT) inhibitor removers. The CDP:AIBN molar ratio applied was 10:1. In a round-bottom flask (25 mL), DMAEMA (0.6 g, 3.8 mmol), OEGMA (1.4 g, 1.5 mmol), AIBN (0.0016 g, 0.01 mmol), and CDP (0.04 g, 0.1 mmol) were dissolved in 1,4-dioxane (10 mL, 20 wt % monomer solution) under magnetic stirring. The flask was sealed with a rubber septum, and the polymerization mixture was degassed via nitrogen bubbling for 20 min. Afterwards, the flask was placed in an oil bath under stirring at 70 °C for 24 h. For the termination of the polymerization, the flask was put directly at −20 °C for 30 min, and then the reaction product was exposed to air. Unreacted monomers and other impurities were removed from the product through dialysis against deionized H_2_O for 3 days. After evaporation of water using a rotary evaporator, the synthesized *co*polymer was isolated and dried in a vacuum oven for 48 h at 25 °C.

### 2.3. Chemical Modification of P(DMAEMA-co-OEGMA) Random Copolymer

P(DMAEMA-*co*-OEGMA) random copolymer was chemically modified through the quaternization of the tertiary amine group of the DMAEMA segments to quaternary ammonium salt, utilizing methyl iodide (CH_3_I) as the quaternization agent. A dilute solution (1.5% *w*/*v*) of the *co*polymer (150 mg, 0.34 mmol) in THF (10 mL) was placed in a 25 mL round bottom flask, under magnetic stirring. Then, an excess of 20% CH_3_I (26 μL, 0.41 mmol) was added. The flask was covered with aluminum foil due to the reactive agent’s sensitivity to intense light. The reaction lasted for 24 h at ambient temperature under continuous stirring. Finally, the solution was collected in a vial and placed in a bath at 60 °C to evaporate the solvent. The modified *co*polymer was then placed in a vacuum oven for 48 h for drying.

### 2.4. Self-Assembly of P(DMAEMA-co-OEGMA) Random Copolymer

P(DMAEMA-*co*-OEGMA) and its modified derivative Q_P(DMAEMA-*co*-OEGMA) were dissolved in distilled water (1 × 10^−3^ g/mL), and the solution was left overnight at ambient temperature in order to reach equilibrium (pH = 7). Their self-assembly behavior in aqueous solution was then studied using light scattering techniques. Regarding the P(DMAEMA-*co*-OEGMA) copolymer, three solutions were prepared at three different pHs (3, 7, 10) due to DMAEMA’s pH-responsive character. The pH of copolymer solution was regulated at pH 3 and 10 by adding the appropriate amount of HCl 0.1 M and NaOH 0.1 M, respectively. Each sample was filtered through hydrophilic PVDF 0.45 µm disposable filters before dynamic light scattering measurements.

### 2.5. Ionic Strength Effects

The effect of ionic strength on the polymer solutions was examined using the DLS technique, with the gradual addition of different amounts of NaCl 1 M solution. After consecutive additions, scattering intensity and hydrodynamic radius changes indicated the polymers’ response to the increasing salt concentration. 

### 2.6. Synthesis of CDs

CD synthesis was conducted according to a standard hydrothermal procedure. Citric acid (9.77 g, 5.5 mmol) and ethylenediamine (3.11 mL, 5 mmol) were dissolved in 100 mL of deionized water in a Teflon liner. The Teflon liner was then placed in an autoclave device and held at 200 °C for 5 h. When the autoclave was cooled at room temperature, the product was filtered under vacuum, using water and a minimum amount of methanol as the solvent. The final product was stored in a vial and dried in a vacuum dryer. 

### 2.7. Complexation of Carbon Dots with Random Copolymers

The P(DMAEMA-*co*-OEGMA)/CD and Q_P(DMAEMA-*co*-OEGMA)/CD complexes were prepared by adding appropriate amounts of CD solution (c = 1 × 10^−4^ g/mL) to an aqueous solution of the respective polymer (c = 1 × 10^−3^ g/mL). The addition was carried out dropwise and under stirring, at ambient temperature. Then, the respective solution was left for 20 min under gentle stirring for the complexes to form and equilibrate. The calculations for the mixtures of the solutions were based on four different mass ratios between the *co*polymers and the CDs. The Pol(+)/CDs(−) ratios used were 5%, 10%, 20%, 50%. The same ratios were applied for the complexes of the modified *co*polymer, Q_Pol(+)/CDs(−). In each case, the amount of polymer was kept constant (5 mL, c = 1 × 10^−3^ g/mL), and the volume of the CDs was changed based on the desired ratio. The final solutions were diluted with distilled water to a final volume of 10 mL. After dilution, the solutions were left under stirring for 10 min, and then the systems were left for one day to equilibrate before any measurements.

### 2.8. Interaction with Iron Ions

The complexes interacted with iron ions (Fe^3+^) in aqueous solution to examine the effect of iron ion (Fe^3+^) concentration on the photophysical properties of complexed CDs, as a preliminary step towards sensing applications. For that purpose, small amounts of Fe^3+^ from an aqueous solution of FeCl_3_ (c = 0.1 mg/mL) were added to portions of complex solutions (1.5 mL). The quantities were calculated so that the concentration of Fe^3+^ in each complex solution was 10, 50, and 100 ppm. The samples were measured using FS spectroscopy, 5 min after the addition.

### 2.9. Characterization Techniques

#### 2.9.1. Size-Exclusion Chromatography (SEC)

Size-exclusion chromatography was performed to determine the molecular weight and dispersity index of the copolymer. A Waters chromatography instrument was used, consisting of a Waters 1515 isocratic pump, a set of three μ-Styragel mixed pore separation columns (pore range 10^2^–10^6^ Å), and a Waters 2414 refractive index detector (equilibrated at 40 °C), and it was controlled using Breeze software (version 2.0). Tetrahydrofuran, containing 5% *v*/*v* triethylamine, at a flow rate of 1 mL/min at 30 °C was used as the eluent. The copolymer was dissolved in the eluent at a concentration of 2 mg/mL. Polystyrene samples of low dispersity (M_w_ = 1200–900,000) were used as standards. 

#### 2.9.2. Proton Nuclear Magnetic Resonance Spectroscopy (^1^H-NMR)

^1^H-NMR spectroscopy was utilized for the verification of the chemical structure and mass composition (wt. %) of P(DMAEMA-*co*-OEGMA), as well as the quaternization degree of its Q_P(DMAEMA-*co*-OEGMA) derivative. ^1^H-NMR spectra were recorded using a Bruker AC 300 FT-NMR spectrometer (Billerica, MA, USA), and tetramethylsilane (TMS) was used as the reference. Samples for NMR measurements were prepared by dissolving 10 mg of the sample in 0.7 mL of deuterated chloroform (CDCl_3_) or deuterated water (D_2_O).

#### 2.9.3. Attenuated Total Reflectance–Fourier Transform Infrared (ATR–FTIR) Spectroscopy

Measurements in the near-infrared region (550–4000 cm^−1^) were performed using a Fourier transform spectrometer (Equinox 55 from Bruker Optics) equipped with a single-bounce ATR diamond accessory (Dura-Samp1IR II from SensIR Technologies, Danbury, CT, USA). Each spectrum was acquired as the average of 64 scans collected at 4 cm^−1^ resolution.

#### 2.9.4. Absorption Spectroscopy (UV-Vis-NIR)

UV-Vis-NIR spectra were recorded using a Perkin–Elmer (Lambda 19, Waltham, MA, USA) UV-Vis-NIR spectrophotometer. CDs’ and complexes’ solutions (3 mL) were placed in quartz cells for the measurements.

#### 2.9.5. Fluorescence Spectroscopy (FS)

Fluorescence spectra were recorded on a Fluorolog-3 JobinYvon-Spex spectrofluorometer (model GL3–21, Kyoto, Japan), using a laser diode as the excitation source (NanoLED, 440 nm, pulse width 100 ps) and a TBX-PMT series UV detector (250–850 nm) from Horiba Jobin Yvon. The micropolarity of the copolymer aggregates in aqueous solution at different pHs was evaluated by utilizing pyrene as the fluorescent probe. Pyrene is sensitive to the polarity of the surrounding environment when residing within the polymer aggregates formed in each case. A quantity of pyrene solution (in acetone, 1 mM) was added to each solution at a ratio of 1 µL/1 mL. The samples were allowed to equilibrate for 24 h (in this period, acetone evaporation takes place) and then measured at ambient temperature.

#### 2.9.6. Dynamic Light Scattering (DLS)

Dynamic light scattering measurements were performed using the ALV/CGS-3 Compact Goniometer System instrument (ALV GmbH, Langen, Germany), equipped with a JDS Uniphase 22 mW He-Ne laser operating at 632.8 nm, connected to a 288-channel ALV-5000/EPP multi-tau digital correlator and an ALV/LSE-5003 light scattering electronic unit for stepper motor drive and limit switch control. The correlation functions were the average of five measurements, and they were analyzed using the cumulants method and the CONTIN algorithm, which provides the distributions for the apparent hydrodynamic radii of species in solution using an inverse Laplace transformation of the autocorrelation function and with the help of the Stokes–Einstein relation. All solutions were filtered through a 0.45 μm porosity hydrophilic PVDF filter prior to measurements for the removal of dust particles. The measurements were conducted at an angle of 90 degrees and a temperature range of 25 to 55 °C controlled by an external bath circulator.

#### 2.9.7. Electrophoretic Light Scattering (ELS)

Zeta potential was measured on a Malvern instrument (Nano Zeta Sizer, Malvern, UK) equipped with a 4 mW He-Ne laser of wavelength λ = 633 nm, which uses a photodiode as a detector, and the scattered radiation was measured at an angle of 173°. Electrophysical measurements to determine the mobility and Zp values of colloids were performed using the LDV (Laser Doppler Velocimetry) technique and the Smoluchowski approach. The reported Zp values are an average of 100 measurements.

#### 2.9.8. Thermogravimetric Analysis (TGA)

Thermogravimetric analysis was performed using a TGA Q500 V20.2 Build 27 instrument by TA (New Castle, DE, USA) in a nitrogen (purity > 99.999%) inert atmosphere.

## 3. Results and Discussion

### 3.1. Synthesis and Molecular Characterization of P(DMAEMA-co-OEGMA) and Its Quaternized Derivative

**Scheme 1:**
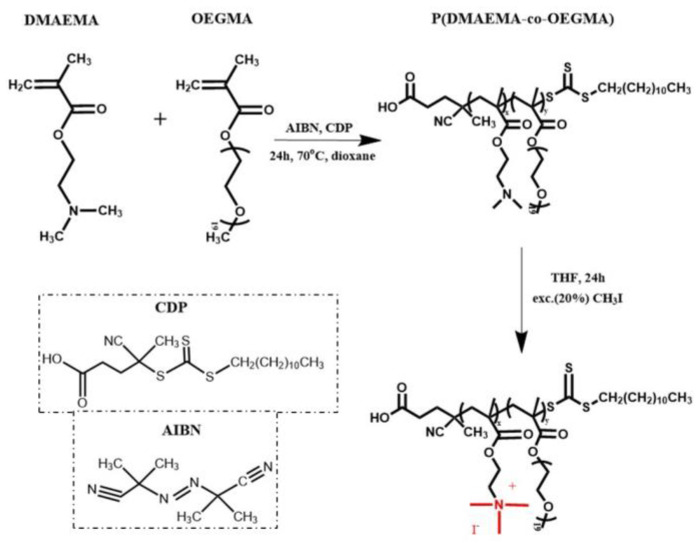
Scheme for P(DMAEMA-*co*-OEGMA) copolymer synthesis via RAFT polymerization and chemical modification of P(DMAEMA-*co*-OEGMA) with methyl iodide (CH_3_I).

Double-hydrophilic poly(2-dimethylaminoethyl methacrylate-*co*-oligo(ethylene glycol) methyl ether methacrylate) was synthesized via a one-step RAFT polymerization, as shown in Scheme 1. Oligo (ethylene glycol) methacrylate with M_n_ = 950 g/mol (19 ethylene oxide units on the side chain) was selected due to its ability to enhance colloidal stability and add shielding properties to the final product. The selected CTA (Chain Transfer Agent) was 4-cyano-4-(dodecylsulfanylthiocarbonyl) pentanoic acid (CDP) due to its compatibility with methacrylic monomers [27]. Size-exclusion chromatography (SEC) was utilized in order to determine the copolymer’s molecular weight and molecular weight distribution. The chromatography analysis (Figure S1) illustrated the success of the polymerization scheme and the efficacy of the purification of the P(DMAEMA-*co*-OEGMA) copolymer through dialysis against deionized water. Successful control is indicated by the molecular weight, which is close to the stoichiometric one, and the low molecular weight distribution obtained, which falls within the range of the standard reported values regarding random copolymers produced through RAFT polymerization. The overall molecular characteristics of the copolymer extracted through SEC are given in Table 1.

Following the synthesis, the P(DMAEMA-*co*-OEGMA) random copolymer was converted into a strong polyelectrolyte with permanent cationic charges, as presented in Scheme 1. Through this chemical modification, the tertiary amine groups of the DMAEMA segments were converted into positively charged quaternary ammonium salts. In this way, a more hydrophilic copolymer was produced, and its responses to pH and temperature changes are expected to significantly weaken.

**Figure 1:**
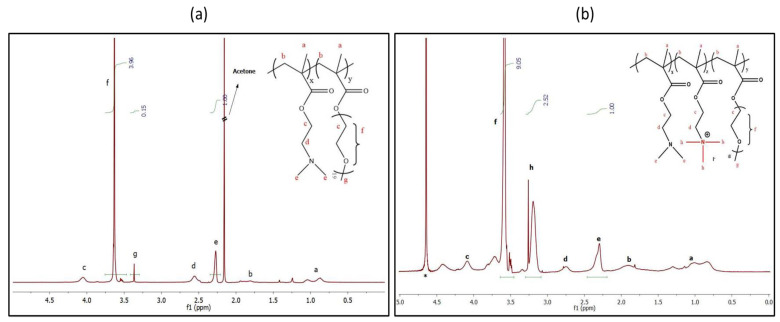
^1^H-NMR spectra for (**a**) P(DMAEMA-*co*-OEGMA) random copolymer in deuterated chloroform (CDCl_3_) * (7.3 ppm, not shown) and (**b**) Q_P(DMAEMA-*co*-OEGMA) copolymer in deuterated water (D_2_O) *.

**Figure 2:**
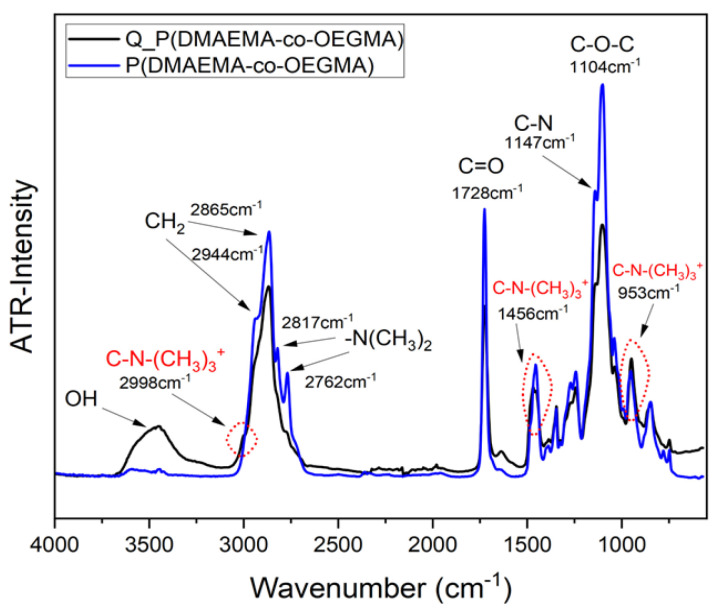
ATR–FTIR spectra of P(DMAEMA-*co*-OEGMA) *co*polymer and Q_P(DMAEMA-*co*-OEGMA) copolymer (black line) in comparison to its precursor (blue line).

The chemical structure and composition of the random copolymer and the modified derivative were identified through ^1^H-NMR (Figure 1) and ATR-FTIR (Figure 2) spectroscopies. As for the ^1^H-NMR spectrum for P(DMAEMA-*co*-OEGMA), as shown in Figure 1a, the chemical composition of each segment was found by estimating the integrals of the characteristic peaks. Specifically, the characteristic peaks of DMAEMA and OEGMA segments are observed at 2.25 ppm (peak e, 6H, -N(CH_3_)_2_-) and 3.36 ppm (peak g, 3H, -CH_3_-), corresponding to the -CH_3_ protons of the tertiary amine group of the DMAEMA segment and the -CH_3_ protons of the side chain methyl of the OEGMA monomer, respectively. Notably, a strong peak is observed around 2.15 ppm, which is possibly attributed to acetone residues. Regarding its modified derivative Q_P(DMAEMA-*co*-OEGMA) (Figure 1b), the characteristic spectral peaks are observed at 2.25 ppm (peak e, 6H, -N(CH_3_)_2_-), 3.36 ppm (peak h, 9H, -N(CH_3_)_3_-), and 3.63 ppm (peak f, 4H, -(CH_2_CH_2_O)_9_-) and belong to the -CH_3_ protons of the quaternary amine group of Q_DMAEMA, the -CH_3_ protons of the tertiary amine group of DMAEMA segment, and the -CH_2_ protons of the ethylene glycol side chain of OEGMA monomer, respectively. As observed in the modified derivative’s spectrum, a quantity of the DMAEMA segment is still observed, meaning that the chemical modification does not take place to the full extent and a small percentage of the DMAEMA monomer is still present. Moreover, the spectrum of the quaternized polyelectrolyte was obtained using deuterium oxide (D_2_O) as the solvent, as it was insoluble in deuterated chloroform (CDCl_3_). On the other hand, the ATR-FTIR spectra provided qualitative results regarding the chemical structures of copolymers and typical functional groups in each case. The spectrum of the modified polyelectrolyte, in comparison to its precursor (Figure 2), shows a number of new, discrete peaks, thus corroborating qualitatively the modification.

### 3.2. Synthesis and Characterization of CDs

**Scheme 2:**
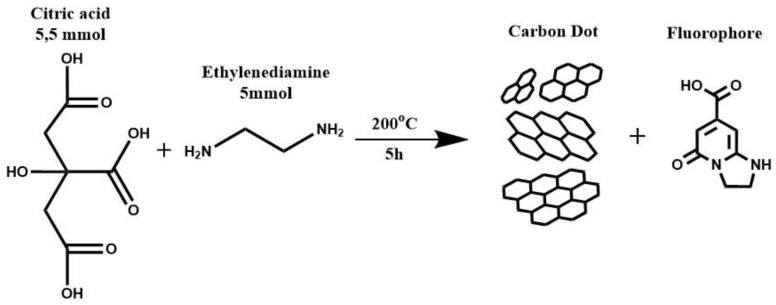
Synthetic conditions for the preparation of citric-acid-based CDs.

CDs were synthesized through a standard hydrothermal protocol that included the reaction of citric acid with ethylenediamine, as shown in Scheme 2 [1]. This synthetic route results in CDs via carbonization of the precursor (ethylenediamine) at 200 °C. In addition, this reaction yields strongly fluorescent 5-oxo-1,2,3,5-tetrahydroimidazo[1,2-α]pyridine-7-carboxylic acid (IPCA), a derivative of citrazinic acid that adds to the CDs’ elevated photoluminescence quantum yield. Moreover, the excess amount of citric acid in this reaction (5.5 mmol) results in the appearance of negative surface charges in the synthesized CDs, due to the excess of carboxyl groups. Structural information regarding the CDs was acquired through ATR-FTIR spectroscopy and TGA analysis (Figure S2). As for their chemical structure, ATR-FTIR spectroscopy (Figure S2a) revealed the presence of characteristic chemical groups of CDs. In more detail, the broad absorption peak at 3323 cm^−1^ is attributed to alcohol (-OH) and amine (-NH_2_) groups, whereas the strong signals at 1639 and 1550 cm^−1^ are ascribed to the C=O and -NH_2_ stretching vibrations, respectively. C=O stretching vibrations originate from α,β unsaturated carboxylic acid moieties present in IPCA fluorophore. In addition, the CD sample was subject to thermogravimetric analysis (TGA), which established the weight loss of the material at increasing temperature due to decomposition of the chemical structures (Figure S2b).

**Figure 3:**
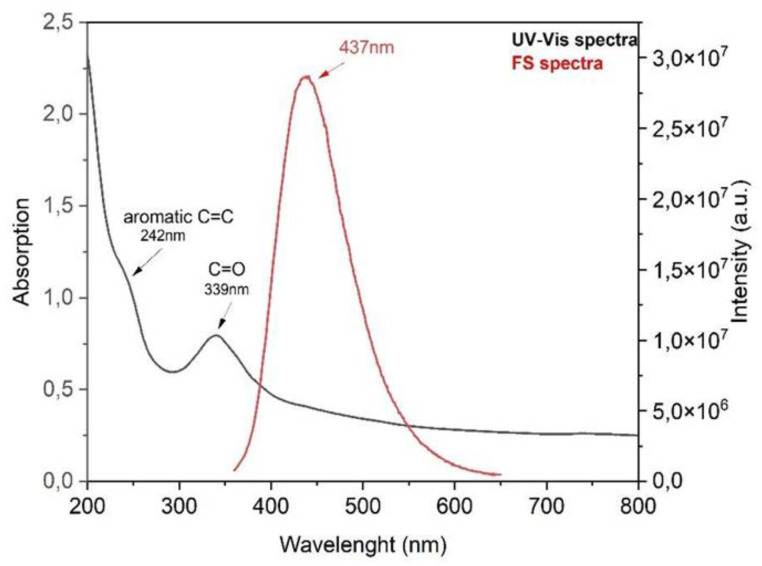
UV-Vis and photoluminescence spectra (FS) for CD aqueous solution.

Further spectroscopic studies revealed the fluorescence properties of the CDs caused by the fluorophore citrazinic acid derivative, IPCA. Figure 3 shows the absorption and photoluminescence spectra, in correlation, obtained through UV-Vis and PL spectroscopies. The absorption spectrum shows a shoulder at 242 nm and a broader peak at 339 nm, assigned to π–π* transitions of sp^2^-hybridized carbon and n–π* transitions at the edge of the carbon lattice, respectively. On the photoluminescence emission spectrum, a high intensity peak is observed at 437 nm, originating from excitation at 340 nm and attributed to n–π* transitions at the CD edge [28]. This strong emissive effect stems from the molecular fluorophore in relation to the citrazinic acid. The excitation area was chosen in correspondence with the absorption spectrum and the strong peak obtained.

In addition, certain parameters, such as the apparent hydrodynamic radius (R_h_), scattered light intensity (I), size dispersity (PDI), and surface charges (Z_p_) of the CD aqueous solution, were determined via dynamic and electrophoretic light scattering measurements. The hydrodynamic radius (R_h_) was found via CONTIN analysis, whereas the size dispersity index (PDI) was determined using the cumulants method. The results of this study are showcased in Table S1 and Figure S3, accordingly. Electrophoretic light scattering measurements certified the strong negative surface charges of the CDs, owing to the excess of citric acid and, hence, carboxyl groups. It is noteworthy that the CDs in aqueous solution demonstrated bimodal size distribution, including a population of larger nanoparticles with a size of 107 nm. Given that CDs acquire sizes below 10 nm, the obtained data indicate that the prepared CDs formed aggregates with low mass (I = 47 kHz) and high heterogeneity (PDI = 0.431) [29] in aqueous solution.

### 3.3. Stimuli Response of Double-Hydrophilic P(DMAEMA-co-OEGMA) and Its Modified Derivative Q_P(DMAEMA-co-OEGMA) in Aqueous Solution

Random copolymers usually succumbed to intramolecular self-folding when added to aqueous solution, thus forming micellar-like nanoaggregates. This behavior is strongly affected by the hydrophobic/hydrophilic ratio as well as the chain length [12]. In order to examine the self-assembly behavior of the polyelectrolytes in response to external stimuli, light scattering studies were conducted on their aqueous solution. In particular, the measurements performed showcase the effect of temperature, pH, and ionic strength changes on the properties of each aqueous copolymer solution.

#### 3.3.1. Thermo-Responsiveness of Double-Hydrophilic P(DMAEMA-*co*-OEGMA) Copolymer

Temperature changes can drastically affect certain characteristics, such as morphological configuration, solubility, and self-assembly behavior, of stimuli-responsive polymers, which usually consist of hydrophilic and hydrophobic segments [18]. Therefore, in such systems, the main study concerns the hydrophilicity changes as a consequence of temperature changes. Polymers capable of phase transition, according to their solubility, might have lower critical solution temperature (LCST), meaning that below this temperature, the system shows increased solubility and homogeneity in water, while induced hydrophobicity and phase separation occur when heating above this critical condition [30]. In this case, the PDMAEMA homopolymer has an LSCT in the range of 40–50 °C, hence showing highly responsive behavior to temperature changes around this temperature range. The phase separation that occurs in such cases is strongly dependent on the molecular weight of the homopolymer and certain conditions, such as the pH and the salt content. On the other hand, the POEGMA homopolymer, with LCST at above 90 °C, provides enhanced hydrophilicity and shielding properties [24,25].

**Figure 4:**
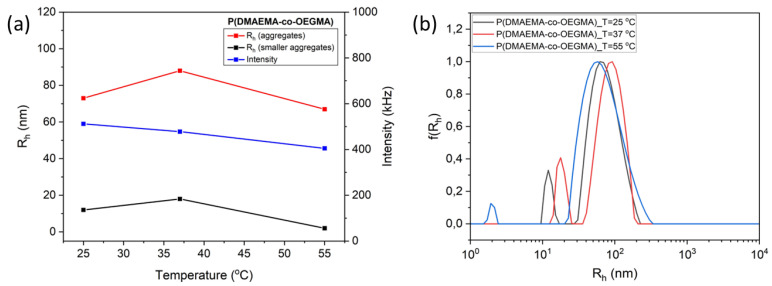
(**a**) Variation in scattering intensity and hydrodynamic radius of P(DMAEMA-*co*-OEGMA) upon temperature changes (θ = 90 ^ο^ and pH = 7) and (**b**) size distributions from CONTIN analysis during temperature increase (from 25 °C to 55 °C) extracted from DLS measurements.

In order to record any phase transitions and increased hydrophobicity, dynamic light scattering measurements were carried out in function with the temperature of the copolymer solution. The obtained results (Figure 4) showcase a relatively weak thermo-responsive behavior of the copolymer system, which is justified by its low content of DMAEMA segments (36%), which is related to the small molecular weight of the PDMAEMA homopolymer. As mentioned before, the DMAEMA component is highly responsive to temperature changes when incorporated in the polymer chain, whereas the dominant OEGMA component adds colloidal stability to the copolymer system. Notably, it is observed that the nanoparticles’ dispersity index takes relatively high values, particularly at 25 °C and 37 °C, indicating the heterogeneity of the existing populations in solution. Populations of smaller sizes, evident in Figure 4b, are attributed to the presence of single chains of the copolymer in the solution or smaller-size aggregates incorporating a small number of chains. Furthermore, as shown in Figure 4a and Table S2, a slight increase in hydrophobicity and, therefore, in hydrodynamic radius is observed at 37 °C (18 nm/88 nm), a temperature near the LCST of PDMAEMA, for both populations. This means that close to this temperature, the above-mentioned copolymer succumbs to further intramolecular self-folding, forming aggregates of slightly increased size. By further increasing the temperature to 55 °C, the sizes decrease (2 nm/67 nm), most likely due to chain shrinkage or the breaking of the aggregates into smaller-sized nanostructures. Moreover, as the temperature gradually increases, scattered intensity values tend to slightly decrease, following the multichain aggregates’ mass reduction.

#### 3.3.2. pH-Responsiveness of Double-Hydrophilic P(DMAEMA-*co*-OEGMA) Copolymer

Polymer systems composed of ionizable segments can be strongly affected by pH changes. As a result, the hydrodynamic volume of the polymer chains undergoes some significant alterations, owing to the rapid charge changes [21]. As a weak cationic polyelectrolyte, PDMAEMA carries tertiary amine groups that are charged depending on the solution’s pH. When the pH is neutral, the amino groups are partly protonated, and thus the polymer chains acquire a relatively hydrophilic character. At lower pH values (pH < 6), dimethylamino groups located on DMAEMA segments are fully protonated, resulting in highly charged polymer nanodomains with high levels of water solubility. On the contrary, the amino groups are deprotonated when the pH is basic (pH > 8), establishing a hydrophobic character for the polymer chains and intensifying the interchain aggregation phenomena in the aqueous solution [12]. Based on these tendencies exhibited by DMAEMA segments, the self-assembly behavior of the P(DMAEMA-*co*-OEGMA) copolymer at various pH values was thoroughly investigated through light scattering techniques and fluorescence spectroscopy.

**Figure 5:**
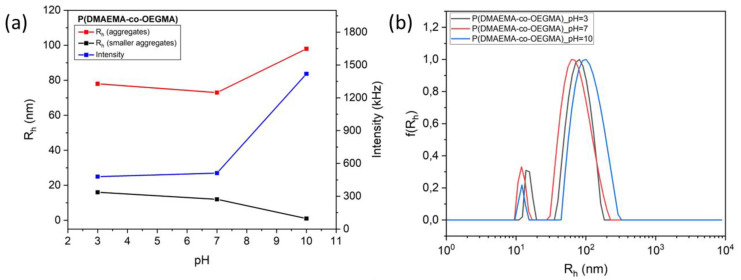
(**a**) Variation in scattered intensity and hydrodynamic radius of P(DMAEMA-*co*-OEGMA) and (**b**) size distributions from CONTIN analysis at pH 3, 7, and 10, extracted from DLS measurements.

The results of the dynamic and electrophoretic light scattering measurements under different pH conditions are summarized in Table S3. At first glance, the copolymer aqueous solution at neutral conditions creates bimodal size distributions, establishing the presence of two distinct populations of different sizes (Figure 5b). It is observed that scattering intensity as well as hydrodynamic radius are significantly increased at pH = 10 (I = 1419 kHz, R_h_ = 98 nm), due to the deprotonation of amino groups of the DMAEMA monomeric units. In this state, hydrophobic interactions prevail in the solution; therefore, multichain aggregates are formed to a larger extent. On the other hand, at acidic and neutral conditions, PDMAEMA performs in a more hydrophilic manner, as the dimethylamino groups of the DMAEMA segments are fully or partially protonated, respectively. Nevertheless, when the pH is changed from neutral to acidic, the hydrodynamic radius of P(DMAEMA-*co*-OEGMA) is slightly increased, as opposed to the scattered intensity that merely decreases (Figure 5a). This rather small difference might be attributed to the somewhat improved homogeneity of the copolymer system (PDI = 0.36) in an acidic environment.

Furthermore, Table S3 displays the zeta-potential values that were determined via ELS measurements and established the charge changes in the macromolecules’ solution. As expected, the protonation/deprotonation of the amino groups in the DMAEMA segments upon pH changes defines the effective surface charge of the nano-assemblies formed. At acidic conditions (pH = 3), the surface charge is positive (ζ_p_ = +17 mV) due to the full protonation of the DMAEMA segments, while, at neutral pH, the dimethylamino groups are partially protonated and the surface charge preserves highly positive values (ζ_p_ = +16 mV). In contrast, the zeta-potential value is negative at pH = 10 (ζ_p_ = −5 mV), as DMAEMA segments are no longer protonated.

The transition from hydrophilic to hydrophobic behavior was also studied through FS spectroscopy by using pyrene as a tracer for the qualitative measurement of the environmental micropolarity. Pyrene is a hydrophobic compound that shows minimal solubility in aqueous solution. Interestingly, information about the polarity of the medium is extracted through the ratio of the first and the third peak on its emission spectrum. Concerning P(DMAEMA-*co*-OEGMA), the hydrophobic character of the system at pH = 10 is exhibited by the decrease in the I_1_/I_3_ ratio (Figure S4), indicating that pyrene is trapped into the produced aggregates (probably within existing hydrophobic nanodomains).

#### 3.3.3. Salt-Induced Responsiveness of P(DMAEMA-*co*-OEGMA) Random Copolymer and Q_P(DMAEMA-*co*-OEGMA) Derivative

As a weak cationic polyelectrolyte, P(DMAEMA-*co*-OEGMA) self-assembly behavior may be affected by the presence of salt in the aqueous solution. Salt ions compete with macromolecules in aqueous solution to bond with water molecules, resulting in the reduction of the solubility of the polymer chains and the further formation of aggregates in solution (*salting out effect*) [23]. In the present study, the self-assembly behavior of the polyelectrolytes’ solution in response to salt addition was investigated upon NaCl (1 M) titration at pH = 7 and ambient temperature through DLS measurements.

**Figure 6:**
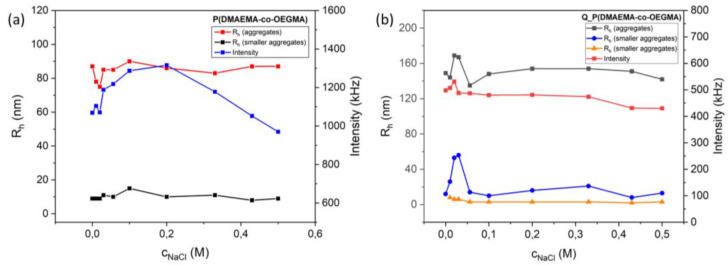
Salt-induced response of scattered intensity and hydrodynamic radius for (**a**) P(DMAEMA-*co*-OEGMA) and (**b**) Q_P(DMAEMA-*co*-OEGMA) random *co*polymer solution, established through DLS measurements at θ = 90^o^ and pH = 7.

Regarding P(DMAEMA-*co*-OEGMA), as observed in Figure 6a, scattered intensity increases during the first seven salt additions (NaCl 1 M), suggesting the system’s tendency for aggregation. However, at salt concentration [NaCl] > 0.2 M, the system mass, indicated by the measured scattered intensity, decreases anew, owing to the tendency towards disintegration of initial aggregates. Moreover, the hydrodynamic radius value marginally decreases upon the first two salt additions, while, beyond this concentration, hydrodynamic radius is increased and then stabilized to the initial value during the next salt additions. This small change in the nanoparticle’s size occurs due to swelling, as the solvent enters their volume and the aggregates become structurally looser during disintegration.

Concerning Q_P(DMAEMA-*co*-OEGMA) (Figure 6b), when dissolved in an aqueous solution (in the absence of salt), it forms aggregates of different sizes (R_h_ = 12/149 nm) and masses (I = 498 kHz) compared to the double-hydrophilic *co*polymer precursor. Therefore, modification through quaternization creates larger aggregates with improved homogeneity (PDI = 0.257). At a salt concentration of [NaCl] < 0.02 M, the scattered intensity slightly increases, accompanied by an increase in the hydrodynamic radius, indicating the further aggregation of the system. Subsequently, the next salt additions ([NaCl] > 0.03 M) cause a decrease in the nanoparticles’ size and mass, as the aggregates presumably somewhat disintegrate. Thereafter, scattered intensity and hydrodynamic radius remain relatively constant.

In summary, upon salt addition, small changes can be observed as far as the scattered intensity (aggregate mass) and hydrodynamic radius of each *co*polymer system are concerned. However, the presence of distinct populations of different sizes is still observed on both occasions. The overall colloid stability of the *co*polymer aggregates in water should be attributed to the high content of OEGMA monomer.

### 3.4. Complexation of CDs with P(DMAEMA-co-OEGMA) and Q_P(DMAEMA-co-OEGMA) Random Copolymers

**Scheme 3:**
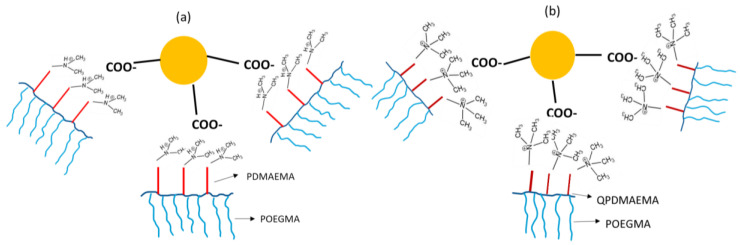
Schematic illustration of the complexation between negatively charged carbon dots and positively charged DMAEMA groups of (**a**) P(DMAEMA-*co*-OEGMA) and (**b**) Q_P(DMAEMA-*co*-OEGMA) random copolymers.

As previously stated, the DMAEMA monomer carries a tertiary amine that is fully protonated under acidic conditions. Therefore, this protonation/deprotonation results in the appearance of positively charged groups along the macromolecular chain. Thus, the complexation of CDs with P(DMAEMA-*co*-OEGMA) random copolymer was based on the electrostatic interactions between the negatively charged CDs and the positively charged DMAEMA segments of each copolymer (Scheme 3a). On the other hand, the modification of P(DMAEMA-*co*-OEGMA) copolymer with methyl iodide resulted in the substitution of the tertiary amine of DMAEMA to a quaternary ammonium salt. Consequently, a strong polyelectrolyte with permanent positive charges was created. In addition, it was deemed necessary to investigate the complexation of the modified copolymer with the CDs and the possible improvement of the properties of the hybrid nano-systems (Scheme 3b). This extended study was conducted at four different mass ratios of CDs (5%, 10%, 20%, and 50%) for each copolymer system.

#### 3.4.1. Physicochemical Characterization of P(DMAEMA-*co*-OEGMA)/CD and Q_P(DMAEMA-*co*-OEGMA)/CD Hybrid Complexes

**Figure 7:**
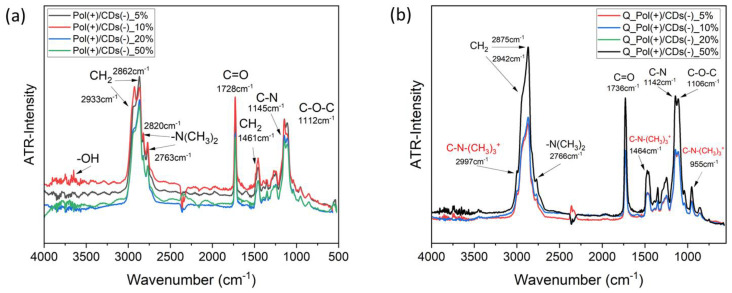
ATR-FTIR spectra of (**a**) P(DMAEMA-*co*-OEGMA)/CD and (**b**) Q_P(DMAEMA-*co*-OEGMA)/CD hybrid complexes.

The obtained hybrid complexes were characterized through FTIR spectroscopy in order to identify the functional groups present on the products and identify possible variations in comparison to the spectra of pure components. As observed in Figure 7, both P(DMAEMA-*co*-OEGMA)/CD (Figure 7a) and Q_P(DMAEMA-*co*-OEGMA)/CD (Figure 7b) systems showcase similar absorption bands to the respective copolymer. It is noteworthy that in comparison to the initial copolymer spectra (Figure 2), a slight shift is observed at 1145 cm^−1^ and 1112 cm^−1^, i.e., the peaks assigned to C-N and C-O-C bond stretching vibrations, respectively. On both occasions, there is a significant decrease in the intensity of the C-O-C peak at the level of the C-N peak. This small alteration may be indicative of the interaction between the CDs and the copolymers.

#### 3.4.2. Thermo-Responsive Behavior of P(DMAEMA-*co*-OEGMA)/CD Hybrid Complexes

As previously discussed, PDMAEMA shows a thermo-responsive behavior, exhibiting LCST in water at 40–50 °C (Section 3.3.1). When the temperature is below the LCST, PDMAEMA c is hydrophilic; hence, the water solubility of the chains is increased. However, at temperatures above the LCST, PDMAEMA is hydrophobic, owing to the fact that hydrogen bonds between PDMAEMA and the aqueous medium are destroyed, and polymer–polymer interactions prevail. Taking that into account, the responsiveness of P(DMAEMA-*co*-OEGMA)/CD hybrid complexes to temperature changes was investigated accordingly.

**Figure 8:**
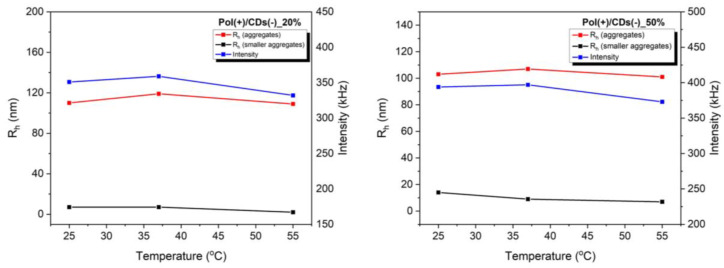
Variation in scattered intensity and hydrodynamic radius of P(DMAEMA-*co*-OEGMA)/CD hybrid complexes upon temperature changes (θ = 90° and pH = 7).

In order to examine the extent of temperature variations’ influence on the physicochemical characteristics of the hybrid complexes, DLS measurements were conducted at neutral pH and different temperatures. The results are summarized in Table S3. The hydrodynamic radius and scattered intensity values show the moderately weak thermo-responsive behavior of the hybrid systems, which corresponds to the behavior of the double-hydrophilic copolymer. Notably, at the 20% and 50% ratios, minor differences are evident regarding the scattered intensity and hydrodynamic radius of the nanoparticles. Namely, the maximum value for both characteristics appears at 37 °C, which is justified by the copolymer’s tendency for agglomeration near the LCST. Figure 8 displays the overall stability of the hybrid complexes for the selected ratios of CDs (20%, 50%) to temperature changes. It is also observed that the dispersity index of the samples decreases at higher concentration of CDs, meaning that the system’s homogeneity is improved and the structure of the complexes is better defined. Moreover, as shown in Figure S5, size distributions of the complexes formed at 20% CDs are not significantly affected by the temperature variations; hence, in this case, the system is quite indifferent to this parameter. Nevertheless, the complexes show wide size distributions due to the increased heterogeneity (PDI = 0.384, pH = 7), regardless of temperature. On the other hand, at 50%, CD heating at 55 °C leads to enhanced homogeneity (PDI = 0.289), as the size distribution of the sample becomes narrower. Peaks observed at smaller sizes are attributed to free copolymer chains, unattached CDs, or maybe complexes of smaller sizes.

#### 3.4.3. pH-Responsive Behavior of P(DMAEMA-*co*-OEGMA)/CD Hybrid Complexes

Based on the response of P(DMAEMA-*co*-OEGMA) to changes in pH and the study discussed in the previous paragraph (Section 3.3.2), DLS and ELS studies were implemented in order to determine the response of the hybrid complexes to pH changes, accordingly. By thoroughly examining the influence of pH changes on the characteristics of the complexes, some interesting conclusions can be drawn about the effects of the complexation with CDs.

**Figure 9:**
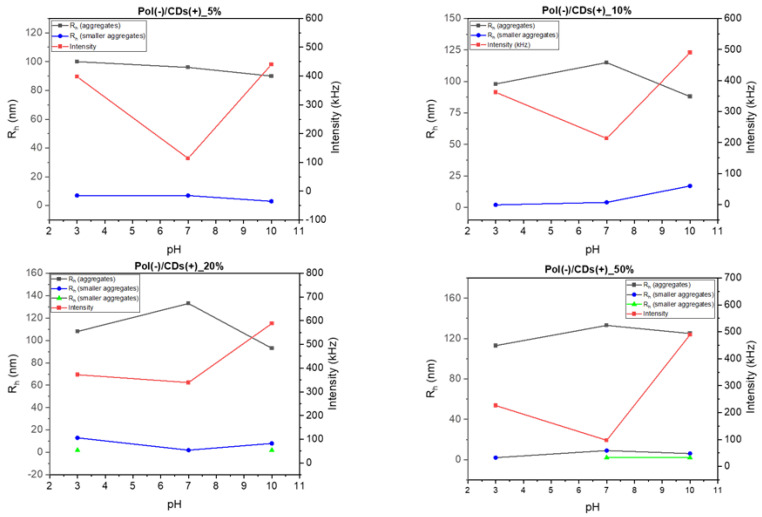
Variation in scattered intensity and hydrodynamic radius of P(DMAEMA-*co*-OEGMA)/CD hybrid complexes at pH 3, 7, and 10 extracted from DLS measurements.

The results of this study, as presented in Table S4, establish a slightly different behavior for the hybrid complexes as a response to pH changes in comparison to the corresponding behavior of the P(DMAEMA-*co*-OEGMA) copolymer. In contrast to the copolymer, which exhibits maximum scattered intensity and hydrodynamic radius values at pH = 10, the complexes display the highest hydrodynamic radius at pH = 7, as under this condition the complexation took place and the complexes were initially formed. In addition, the size of the nanostructures decreases at pH = 3, probably due to the increased solubility of the copolymer. A similar decrease is also seen at pH 10, where the DMAEMA units are deprotonated, thus limiting the presence of positive charges required for the complexation. As for the scattering intensity, its maximum value is found at pH = 10, due to the presence of both complexes already formed and free copolymer chains that tend to create aggregates due to hydrophobic interactions amongst them. Interestingly, the scattering intensity is also relatively increased at pH = 3, most likely due to the presence of an increased number of charged groups of the DMAEMA segments available for complexation with the CDs. Figure 9 displays the overall tendencies of scattered intensity and hydrodynamic radius. Populations with smaller sizes are attributed to single copolymer chains unattached to CDs or complexes of smaller size (Figure S6).

Moreover, ELS studies were carried out to determine the surface charges appearing on the hybrid complexes. It is noteworthy that at the complexation pH (pH = 7), the charge for the complexes at 5% and 10% CD ratios was close to 0, as the compounds’ individual charges tend to cancel out.

#### 3.4.4. Salt-Induced Responsiveness of P(DMAEMA-*co*-OEGMA)/CD and Q_P(DMAEMA-*co*-OEGMA)/CD Hybrid Complexes

**Figure 10:**
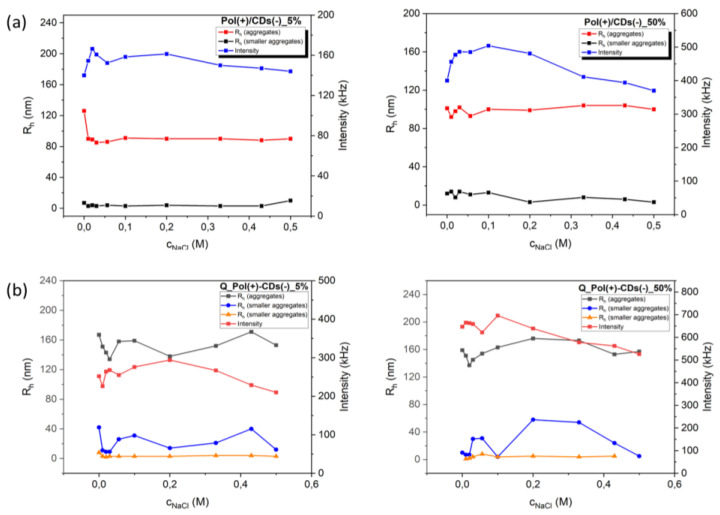
Salt-induced changes in scattered intensity and hydrodynamic radius for (**a**) P(DMAEMA-*co*-OEGMA)/CD and (**b**) Q_P(DMAEMA-*co*-OEGMA)/CD hybrid complexes established through DLS measurements at θ = 90° and pH = 7.

The effect of ionic strength on the solutions of the hybrid complexes was investigated through DLS measurements for both sets of complexes. As for the P(DMAEMA-*co*-OEGMA)/CD hybrid complexes, the hydrodynamic radius and scattering intensity values displayed insignificant variations during the increase in salt concentration (Figure 10a). Hence, changes of little importance were noticed regarding the properties of the nanoparticles.

Concerning Q_P(DMAEMA-*co*-OEGMA)/CD hybrid complexes (Figure 10b), the study of the effect of salt concentration on their solutions was deemed useful, as QPDMAEMA behaves as a strong cationic polyelectrolyte. In total, no significant changes in the size or mass of the complexes were observed, with the exception of those at the 50% ratio of CDs. In this case, a minor decrease in scattered intensity was detected after the consecutive additions, probably due to breakdown of the hybrid nanostructures. The reasons for this behavior are unclear. In addition, two smaller-sized populations are also present, attributed to free chains or smaller hybrid complexes.

#### 3.4.5. Physicochemical Characterization of P(DMAEMA-*co*-OEGMA)/CD and Q_P(DMAEMA-*co*-OEGMA)/CD Hybrid Complexes via Spectroscopic Techniques

**Figure 11:**
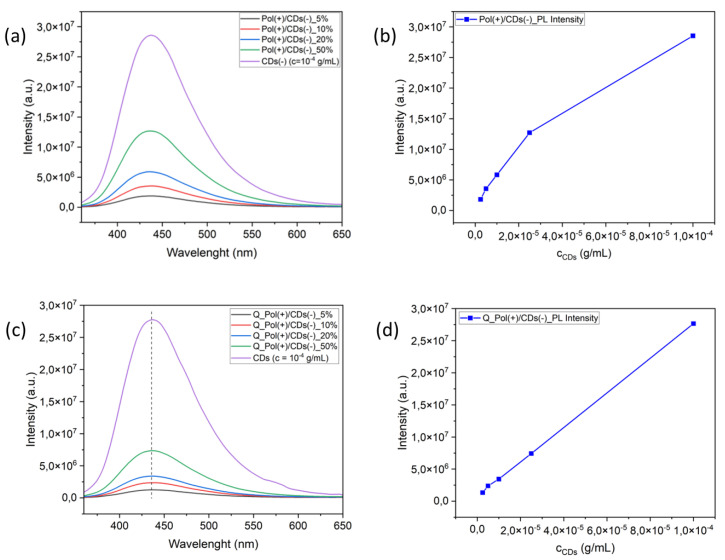
Photoluminescence spectra for (**a**) P(DMAEMA-*co*-OEGMA)/CD and (**c**) Q_P(DMAEMA-*co*-OEGMA)/CD hybrid complexes, respectively. Fluorescence diagram as a function of CDs’ concentration in the (**b**) P(DMAEMA-*co*-OEGMA)/CD and (**d**) Q_P(DMAEMA-*co*-OEGMA)/CD hybrid complexes.

Correspondingly, the contribution of the CDs to the photoluminescence of the complexes is depicted in the FS spectra (Figure 11). As anticipated, the fluorescence intensity of the samples increases in an almost linear way by increasing the concentration of the CDs. In comparison, Q_P(DMAEMA-*co*-OEGMA)/CD hybrid complexes showed significantly lower fluorescence intensity, probably due to the presence of ammonium/iodine ions, which may act as quenchers of fluorescence.

### 3.5. Interaction of Hybrid Copolymer/CD Complexes with Iron Ions

**Figure 12:**
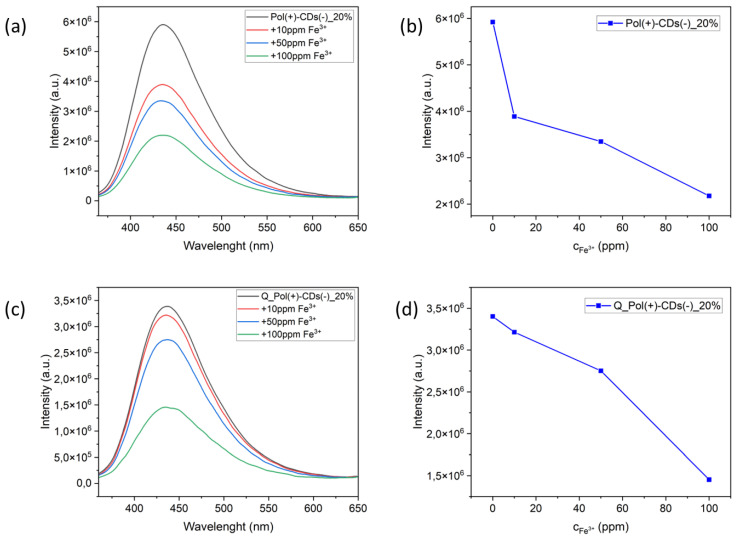
Plots of fluorescence intensity of P(DMAEMA-*co*-OEGMA)/CD (**a**,**b**) and Q_P(DMAEMA-*co*-OEGMA)/CD (**c**,**d**) hybrid complexes as a function of Fe^3+^ concentration.

Lastly, the interest of this study focused on the potential interaction of the obtained hybrid complexes with metal ions in solution. The photoluminescence properties and characteristic functional groups on the surface of CDs allow for the detection of biologically relevant metal ions through resonance [31]. Based on the photoluminescence profile that can be affected by surface group changes, fluorescent CDs can become high-performance nanosensors via various synthetic routes, which include electrochemical etching, microwave and ultrasonic shearing, and pyrolysis. Ref. [32] In this context, the interaction of the prepared hybrid complexes with ferric ions was investigated. Two systems were selected, the hybrid complexes P(DMAEMA-*co*-OEGMA)/CD and Q_P(DMAEMA-*co*-OEGMA)/CD, at ratios of 20% in CDs based on the colloidal stability of complexes over time (Figure S7). Small amounts of a dilute solution of FeCl_3_ were added to the solutions of the hybrid complexes, and, afterwards, the samples were studied through FS spectroscopy. The results of these measurements (Figure 12) show that increased iron concentration in the aqueous solution results in gradually lower fluorescence intensity. Thus, the copolymer/CD complexes can be considered useful for the detection of metal ions in aqueous solution.

## 4. Conclusions

Double-hydrophilic P(DMAEMA-*co*-OEGMA) random copolymer, as well as its Q_P(DMAEMA-*co*-OEGMA) cationic derivative, were successfully used for complexation with negatively charged CDs via electrostatic interactions, leading to novel hybrid nanostructures. Stimuli-responsive P(DMAEMA-*co*-OEGMA) was synthesized via a one-step RAFT polymerization, and then it was molecularly characterized through SEC and ^1^H-NMR. The obtained hybrid complexes displayed similar fluorescence abilities to the CDs. Moreover, light scattering measurements (DLS, ELS) established the formation of slightly larger and more stable nanostructures, with a different response to external stimuli. Based on their fluorescence properties, the hybrid complexes were utilized for the detection of iron ions in aqueous solution, presenting promising results that can contribute to future applications in bioimaging and environmental monitoring.

## Figures and Tables

**Table 1:** Molecular characteristics of P(DMAEMA-*co*-OEGMA) double-hydrophilic random *co*polymer and its modified derivative.

Sample	M_w_ (g/mol) (×10^4^) ^a^	M_w_/M_n_ ^a^	wt. % DMAEMA ^b^	wt. % QDMAEMA ^b^	wt. % OEGMA ^b^
P(DMAEMA-*co*-OEGMA)	1.52	1.19	36	-	64
Q_P(DMAEMA-*co*-OEGMA)	2.02	-	8	25	67

^a^ Determined through SEC. ^b^ Determined through ^1^H-NMR.

## Data Availability

Data will be made available upon request.

## Supplementary Materials

The following supporting information can be downloaded at: https://www.mdpi.com/article/10.3390/ma17102462/s1, Figure S1. SEC chromatogram of P(DMAEMA-*co*-OEGMA) copolymer; Figure S2. (a) FTIR spectrum and (b) TGA thermogram for synthesized CDs; Table S1. Physicochemical characteristics of CDs aqueous solution at neutral pH; Figure S3. Size distributions from CONTIN for the CD aqueous solution; Table S2. Physicochemical features of double-hydrophilic P(DMAEMA-*co*-OEGMA) random copolymer at neutral pH and at various temperatures; Table S3. Physicochemical features of double- hydrophilic P(DMAEMA-*co*-OEGMA) random copolymer at pH 3, 7, and 10; Figure S4. Pyrene fluorescence emission spectrum for P(DMAEMA-*co*-OEGMA) aqueous solution, at various pH values; Table S3. Physicochemical features of P(DMAEMA-*co*-OEGMA)/CD hybrid complexes, at neutral pH and various temperatures; Figure S5. Size distributions from CONTIN analysis of P(DMAEMA-*co*-OEGMA)/CD hybrid complexes during temperature increase (from 25 °C to 55 °C) extracted from DLS measurements; Table S4. Physicochemical features of P(DMAEMA-*co*-OEGMA)/CD hybrid complexes at pH 3, 7, and 10; Figure S6. Size distributions from CONTIN analysis of P(DMAEMA-*co*-OEGMA)/CD hybrid complexes at pH 3, 7, and 10 extracted from DLS measurements; Figure S7. Plots from colloidal stability studies of (a) scattered intensity and (b) hydrodynamic radius of P(DMAEMA-*co*-OEGMA)/CD hybrid complexes over time.

## References

[1] Schneider J., Reckmeier C.J., Xiong Y., Von Seckendorff M., Susha A.S., Kasák P., Rogach A.L. (2017). Molecular Fluorescence in Citric Acid-Based Carbon Dots. J. Phys. Chem. C.

[2] Wu C., Zheng Y., Wang W., Liu Y., Yu J., Liu Y. (2022). Phase Behavior and Aggregate Transition Based on Co-Assembly of Negatively Charged Carbon Dots and a pH-Responsive Tertiary Amine Cationic Surfactant. Langmuir.

[3] Adam G.O., Sharker S.M., Ryu J.H. (2022). Emerging Biomedical Applications of Carbon Dot and Polymer Composite Materials. Appl. Sci..

[4] Kumar A., Narayanan S.S., Thapa B.S., Pandit S., Pant K., Mukhopadhyay A.K., Peera S.G. (2022). Application of Low-Cost Plant-Derived Carbon Dots as a Sustainable Anode Catalyst in Microbial Fuel Cells for Improved Wastewater Treatment and Power Output. Catalysts.

[5] Pham T.H., Aelterman P., Verstraete W. (2009). Bioanode Performance in Bioelectrochemical Systems: Recent Improvements and Prospects. Trends Biotechnol..

[6] Rodrigues J., Pereira S.O., Zanoni J., Rodrigues C., Brás M., Costa F.M., Monteiro T. (2022). ZnO Transducers for Photoluminescence-Based Biosensors: A Review. Chemosensors.

[7] Ganguly S., Das P., Banerjee S., Das N.C. (2019). Advancement in Science and Technology of Carbon Dot-Polymer Hybrid Composites: A Review. Funct. Compos. Struct..

[8] Zulfajri M., Sudewi S., Ismulyati S., Rasool A., Adlim M., Huang G.G. (2021). Carbon Dot/Polymer Composites with Various Precursors and Their Sensing Applications: A Review. Coatings.

[9] Ring W., Mita I., Jenkins A.D., Bikales N.M. (1986). Source-Based Nomenclature for Copolymers (Recommendation 1985). Polym. Sci. USSR.

[10] Moad G., Rizzardo E., Thang S.H. (2008). Radical Addition–Fragmentation Chemistry in Polymer Synthesis. Polymer.

[11] Boyer C., Bulmus V., Davis T.P., Ladmiral V., Liu J., Perrier S. (2009). Bioapplications of RAFT Polymerization. Chem. Rev..

[12] Vardaxi A., Pispas S. (2023). Stimuli-Responsive Self-Assembly of Poly(2-(Dimethylamino)Ethyl Methacrylate-Co-(Oligo Ethylene Glycol)Methacrylate) Random Copolymers and Their Modified Derivatives. Polymers.

[13] Kimura Y., Terashima T., Sawamoto M. (2017). Self-Assembly of Amphiphilic Random Copolyacrylamides into Uniform and Necklace Micelles in Water. Macro Chem. Phys..

[14] Pham D.T., Chokamonsirikun A., Phattaravorakarn V., Tiyaboonchai W. (2021). Polymeric Micelles for Pulmonary Drug Delivery: A Comprehensive Review. J. Mater. Sci..

[15] Nabiyan A., Max J.B., Schacher F.H. (2022). Double Hydrophilic Copolymers—Synthetic Approaches, Architectural Variety, and Current Application Fields. Chem. Soc. Rev..

[16] Van Durme K., Rahier H., Van Mele B. (2005). Influence of Additives on the Thermoresponsive Behavior of Polymers in Aqueous Solution. Macromolecules.

[17] Schmaljohann D. (2006). Thermo- and pH-Responsive Polymers in Drug Delivery. Adv. Drug Deliv. Rev..

[18] Wei M., Gao Y., Li X., Serpe M.J. (2017). Stimuli-Responsive Polymers and Their Applications. Polym. Chem..

[19] Gandhi A., Paul A., Sen S.O., Sen K.K. (2015). Studies on Thermoresponsive Polymers: Phase Behaviour, Drug Delivery and Biomedical Applications. Asian J. Pharm. Sci..

[20] Liu F., Urban M.W. (2010). Recent Advances and Challenges in Designing Stimuli-Responsive Polymers. Prog. Polym. Sci..

[21] Tonge S.R., Tighe B.J. (2001). Responsive Hydrophobically Associating Polymers: A Review of Structure and Properties. Adv. Drug Deliv. Rev..

[22] Cinay G.E., Erkoc P., Alipour M., Hashimoto Y., Sasaki Y., Akiyoshi K., Kizilel S. (2017). Nanogel-Integrated pH-Responsive Composite Hydrogels for Controlled Drug Delivery. ACS Biomater. Sci. Eng..

[23] Mintzer M.A., Simanek E.E. (2009). Nonviral Vectors for Gene Delivery. Chem. Rev..

[24] Mohammadi M., Salami-Kalajahi M., Roghani-Mamaqani H., Golshan M. (2017). Effect of Molecular Weight and Polymer Concentration on the Triple Temperature/pH/Ionic Strength-Sensitive Behavior of Poly(2-(Dimethylamino)Ethyl Methacrylate). Int. J. Polym. Mater. Polym. Biomater..

[25] Lutz J.-F., Hoth A. (2006). Preparation of Ideal PEG Analogues with a Tunable Thermosensitivity by Controlled Radical Copolymerization of 2-(2-Methoxyethoxy)Ethyl Methacrylate and Oligo(Ethylene Glycol) Methacrylate. Macromolecules.

[26] Vassiliadou O., Chrysostomou V., Pispas S., Klonos P.A., Kyritsis A. (2021). Molecular Dynamics and Crystallization in Polymers Based on Ethylene Glycol Methacrylates (EGMAs) with Melt Memory Characteristics: From Linear Oligomers to Comb-like Polymers. Soft Matter.

[27] Moad G. (2017). RAFT Polymerization to Form Stimuli-Responsive Polymers. Polym. Chem..

[28] Sudolská M., Dubecký M., Sarkar S., Reckmeier C.J., Zbořil R., Rogach A.L., Otyepka M. (2015). Nature of Absorption Bands in Oxygen-Functionalized Graphitic Carbon Dots. J. Phys. Chem. C.

[29] Cui L., Ren X., Sun M., Liu H., Xia L. (2021). Carbon Dots: Synthesis, Properties and Applications. Nanomaterials.

[30] Ward M.A., Georgiou T.K. (2011). Thermoresponsive Polymers for Biomedical Applications. Polymers.

[31] Skaltsas T., Goulielmaki M., Pintzas A., Pispas S., Tagmatarchis N. (2017). Carbon Quantum Dots/Block Copolymer Ensembles for Metal-Ion Sensing and Bioimaging. J. Mater. Chem. B.

[32] Zhang Z., Pan Y., Fang Y., Zhang L., Chen J., Yi C. (2016). Tuning Photoluminescence and Surface Properties of Carbon Nanodots for Chemical Sensing. Nanoscale.

