# Supplementary material for: Hybrid Nanoparticles from Random Polyelectrolytes and Carbon Dots
Source: Materials (Basel). 2024 May 20;17(10):2462. doi: 10.3390/ma17102462 (PMC11123412; doi:10.3390/ma17102462)
Supplement: Supplementary file 1 [file materials-17-02462-s001.zip › materials-2966183-supplementary.pdf]

materials MDPI

Supplementary Materials

Hybrid Nanoparticles from Random Polyelectrolytes and Carbon Dots

Sophia Theodoropoulou [1,2], Antiopi Vardaxi [1], Antonia Kagkoura [1], Nikos Tagmatarchis [1,*] and Stergios Pispas [1,*]

1 Theoretical and Physical Chemistry Institute, National Hellenic Research Foundation, 48 Vasileos Konstantinou Avenue, 11635 Athens, Greece; sophiatheodor97@gmail.com (S.T.); avardaxi@eie.gr (A.V.); akagkoura@eie.gr (A.K.)
2 Department of Chemistry, National and Kapodistrian University of Athens, Panepistimioupolis, Zografou, 15771 Athens, Greece
* Correspondence: tagmatar@eie.gr (N.T.); pispas@eie.gr (S.P.)

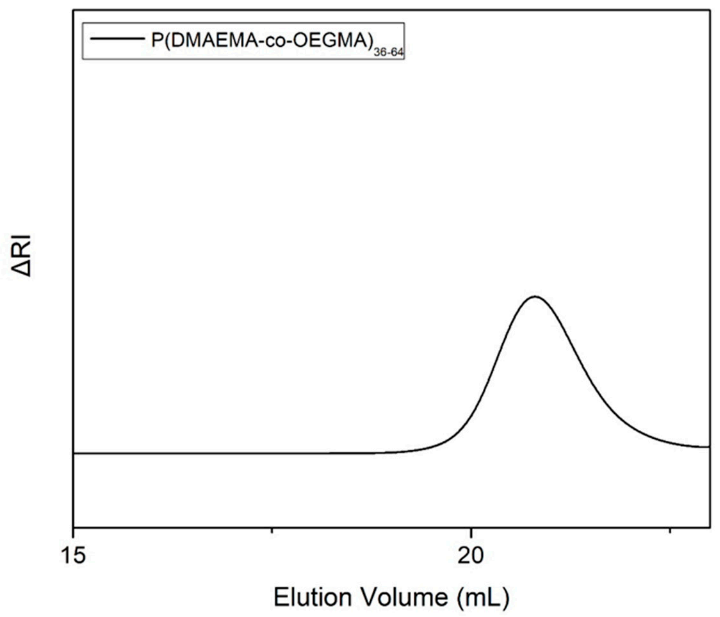

Figure S1. SEC chromatogram of P(DMAEMA-co-OEGMA) copolymer.

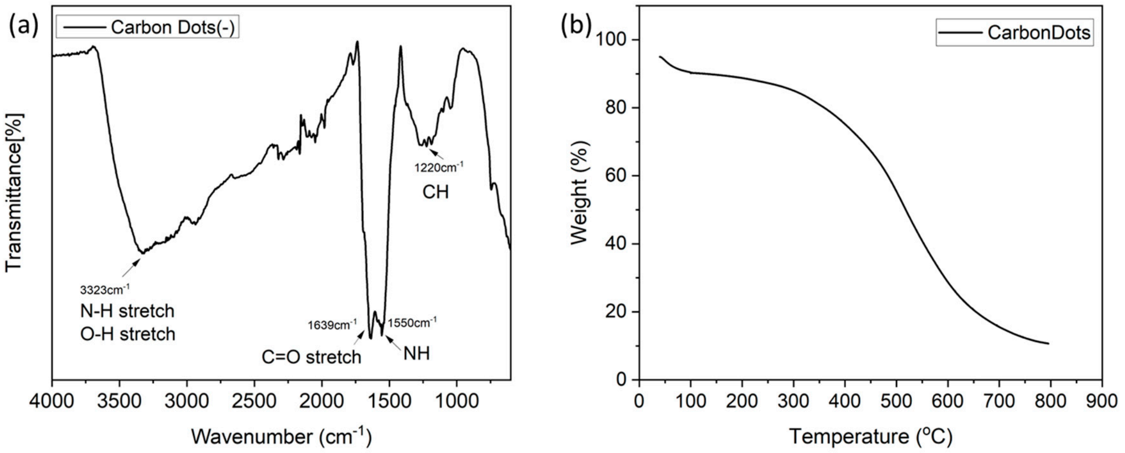

Figure S2. (a) FTIR spectrum and (b) TGA thermogram for synthesized CDs.

Table S1. Physicochemical characteristics of CDs aqueous solution at neutral pH.

Sample	I (kHz)	PDI	R_h (nm)	Z_p (mV)
CDs	47	0.43	1 / 107	-29

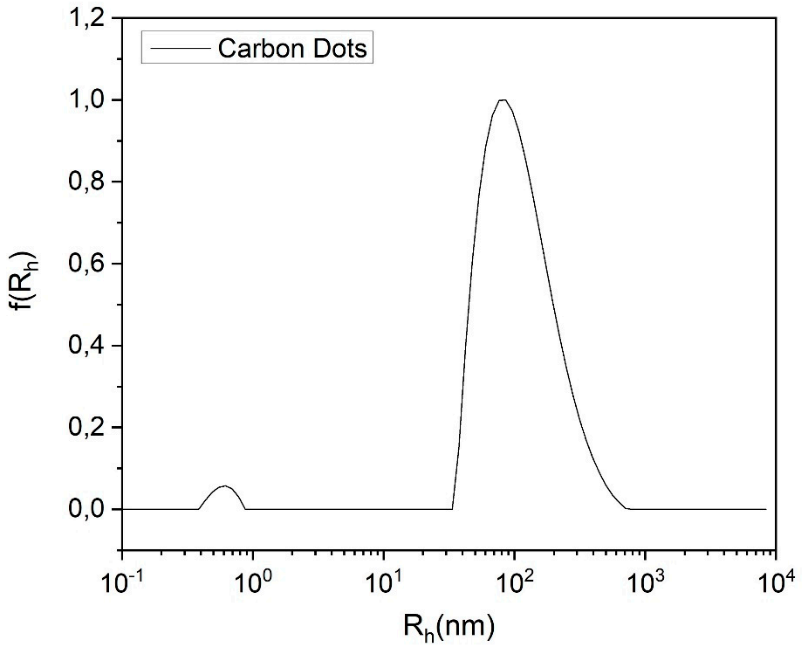

Figure S3. Size distributions from CONTIN for the CDs aqueous solution.

Table S2. Physicochemical features of double hydrophilic P(DMAEMA-co-OEGMA) random *co*polymer at neutral pH and at various temperatures.

Sample	Temperature (°C)	I (kHz) [a]	R_h (nm) [a]	PDI [a]
P(DMAEMA-co-OEGMA	25	512	12 / 73	0.42
	37	478	18 / 88	0.43
	55	405	2 / 67	0.38

[a] Determined by DLS measurements at θ = 90° and pH = 7. The different R_h values correspond to two different populations observed in the aqueous solutions.

Table S3. Physicochemical features of double hydrophilic P(DMAEMA-co-OEGMA) random copolymer at pH 3, 7 and 10.

Sample	pH	I (kHz) [a]	R_h (nm) [a]	PDI [a]	Z_p (mV) [b]
P(DMAEMA-co-OEGMA)	3	479	16 / 78	0.36	+17
	7	512	12 / 73	0.42	+16
	10	1419	1 / 98	0.35	-5

[a] Determined by DLS measurements at θ = 90° and T = 25 °C. The different R_h values correspond to two different populations observed in the aqueous solution. [b] Determined by ELS.

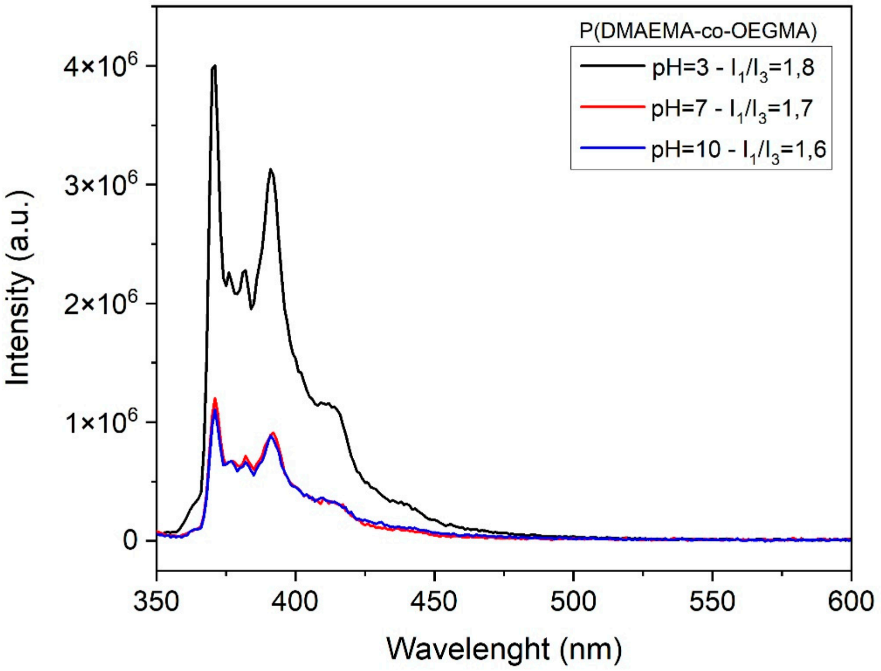

Figure S4. Pyrene fluorescence emission spectrum for P(DMAEMA-co-OEGMA) aqueous solution, at various pH values.

Table S3. Physicochemical features of P(DMAEMA-co-OEGMA) / CDs hybrid complexes, at neutral pH and various temperatures.

Complex	T (°C)	I (kHz) [a]	PDI [a]	R_h (nm) [a]
Pol(+)/CDs(-)_5%	25	140	0.382	7 / 126
	37	133	0.371	2 / 93
	55	127	0.409	2 / 98
Pol(+)/CDs(-)_10%	25	172	0.456	3 / 140
	37	150	0.449	3 / 139

	55	220	0.344	4 / 102
Pol(+)/CDs(-)_20%	25	351	0.376	7 / 110
	37	359	0.384	7 / 119
	55	332	0.373	2 / 109
Pol(+)/CDs(-)_50%	25	394	0.291	14 / 103
	37	397	0.285	9 / 107
	55	373	0.289	7 / 101

[a] Determined by DLS measurements at θ = 90° and T = 25 °C. The different R_h values correspond to two different observed populations in the aqueous solution.

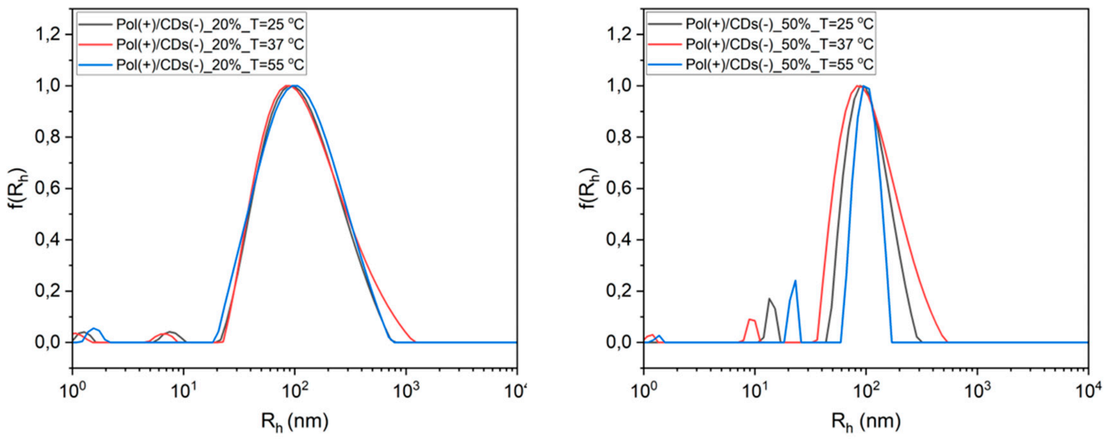

Figure S5. Size distributions from CONTIN analysis of P(DMAEMA-co-OEGMA) / CDs hybrid complexes, during temperature increase (from 25 °C to 55 °C) extracted from DLS measurements.

Table S4. Physicochemical features of P(DMAEMA-co-OEGMA) / CDs hybrid complexes at pH 3, 7 and 10.

Complex	pH	I (kHz) [a]	PDI [a]	R_h (nm) [a]	Z_p (mV) [b]
Pol(+)/CDs(-)_5%	3	398	0.296	7/100	+15.5
	7	114	0.409	7/96	+2.39
	10	440	0.24	3/90	-25.5
Pol(+)/CDs(-)_10%	3	362	0.236	2/98	+16.2
	7	213	0.386	4/115	+0.84
	10	490	0.297	2/17/88	-28
Pol(+)/CDs(-)_20%	3	372	0.307	2/13/108	+37.3
	7	339	0.338	2/133	+6.31
	10	588	0.273	2/8/93	-34.3
Pol(+)/CDs(-)_50%	3	226	0.495	2/113	+29.9
	7	139	0.483	2/161	+10.3
	10	489	0.36	2/6/125	-32.8

[a] Determined by DLS measurements at θ = 90° and T = 25 °C. The different R_h values correspond to two different observed populations in the aqueous solution. [b] Determined by ELS.

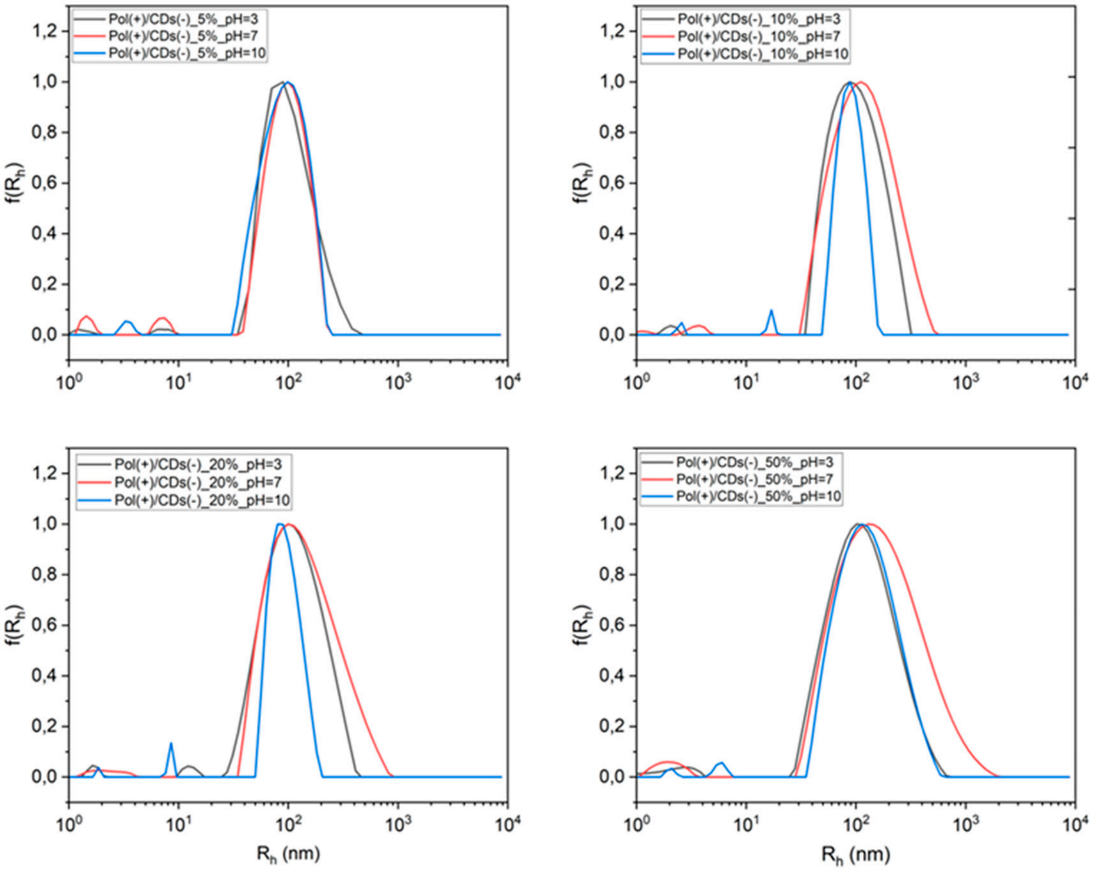

Figure S6. Size distributions from CONTIN analysis of P(DMAEMA-co-OEGMA) / CDs hybrid complexes at pH 3, 7 and 10, extracted from DLS measurements.

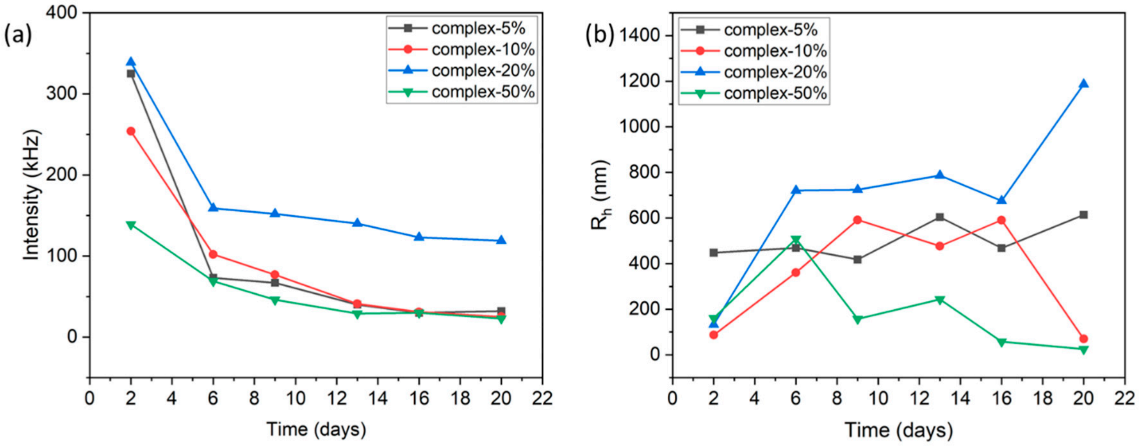

Figure S7. Plots from colloidal stability studies of (a) scattered intensity and (b) hydrodynamic radius of P(DMAEMA-co-OEGMA) / CDs hybrid complexes over time.
